# Duration biased distribution of clinical and immunological phenotypes in active SLE

**DOI:** 10.3389/fimmu.2022.1044184

**Published:** 2022-12-14

**Authors:** Qingran Yan, Bei Liu, Minjie Yang, Qianqian Li, Jieying Wang, Ting Li, Liangjing Lu

**Affiliations:** ^1^ Department of Rheumatology, Ren Ji Hospital, Shanghai Jiao Tong University School of Medicine, Shanghai, China; ^2^ BuXin Community Health Service Center , The People’s Hospital of Baoan Shenzhen, Shenzhen, China; ^3^ Clinical Center for Investigation, Ren Ji Hospital, Shanghai Jiao Tong University School of Medicine, Shanghai, China

**Keywords:** long-established lupus, disease duration, B cell, CD8+ T cell, serological markers

## Abstract

**Introduction:**

This study is aimed to map the clinical and immunological features of active lupus patients with different disease duration.

**Methods:**

For clinical phenotype analysis, we enriched eligible medical records with active SLE (SLEDAI-2k≥8) from the Renji Lupus registry, a single-center database of hospitalized SLE patients with standard care, which covered national-wide patients. Patients with repeated hospitalization records in this enrichment were analyzed longitudinally as validation for the cross-sectional study above.

**Results:**

We enriched a total of 1313 eligible records on active SLE (SLEDAI-2k≥8) for cross-sectional analysis. Stratified into four groups by a 5-year interval of disease duration, these active SLE patients showed a significantly shifting clinical phenotype along with the duration (ascending nephritis, pulmonary hypertension and descending fever, cutaneous symptoms, arthritis, and neuropsychiatric manifestations), especially in stratifications with disease onset age ≤ 45 years old. A longitudinal analysis of 55 patients with repeated hospitalizations for active lupus showed a similar trend. In the cross-sectional study of 222 records with full information on serology and lymphocyte subsets, peripheral B cell proportion, anti-dsDNA antibody, and serum IgG/IgM negatively correlated with duration, while CD8+ T cell proportion was positively correlated (P values, 0.029-4.8×10^-17^), which were supported by the sensitivity analysis in patient subgroups according to disease onset age and recent treatment. Multivariate linear regression identified duration as the only significant associator with both B cell and CD8+ T cell proportion (P values, 8.9×10^-8^ and 7.6×10^-5^, respectively). These duration biased immune phenotypes were highly consistent with the longitudinal observation in 14 patients with repeated hospitalizations.

**Conclusions:**

Both clinical and immunological features of active SLE are significantly duration biased distributed, which merits further investigations in the evolution of SLE pathogenesis.

## Introduction

Systemic Lupus erythematosus (SLE) is a chronic and fluctuant autoimmune disease (1). Present studies on lupus flare pay tremendous attention to the prediction and prevention of flare. In these studies, flares are defined as a general increase in disease activity or hospitalization, with no consideration of specific organ involvement (2–4), which may affect the prevention strategy. Description of organ involvement for established SLE patients remains preliminary and is in short supply.

Pathogenic lymphocyte subsets and their dysfunction are an important part of the pathogenesis study of SLE. However, due to the substantial impact of various treatments on lymphocytes, most in-depth studies have been limited to naive patients (5). For established patients who need therapies demanding more cautious selection, there are few convincing studies to clarify their pathogenic mechanisms. To achieve this goal, the first step would be a comprehensive description of the patient immune features upon different disease courses. In the current study, we tried to make such a description in a population with unified active SLE.

## Materials and methods

### Patients

This is a single-center study based on the Renji Lupus registry, a registry study of hospitalized SLE patients with standard care in Renji Hospital, Shanghai Jiaotong university, school of medicine since 2013. This database was constructed *via* natural language recognition and extraction enhanced by artificial intelligence, with organized information on patient medical history, treatment, and lab tests. Patients would be enrolled if their diagnosis of SLE was confirmed by at least two rheumatology consultant physicians and received standard care (except the hospitalizations specifically for intravenous therapy, e.g., cyclophosphamide or rituximab). Patients with overlap syndromes would not be included in this registry. Written informed consent was obtained from each study participant. The study was conducted in accordance with the Declaration of Helsinki and was approved by the Ethics Committee of Renji Hospital, Shanghai, China (ID: 2013-126).

We selected records dated from 2013 to 2019, ruling out the impact of COVID-19. A record that fulfilled the following criteria would be defined as eligible: 1) with enough information on the clinical features of 2019 EULAR/ACR SLE criteria, 2) with enough information to assess SLEDAI-2k, and 3) the patient should be clinically active disease defined as a SLEDAI-2k score of ≥ 8. All SLEDAI were scored within the first three days of hospitalization as a routine clinical practice in our center. Records with an obvious infection would be ruled out. Patients with positive microbiological evidence or continuous administration of intravenous antibiotics for more than five days were defined as infected.

### Definitions of clinical features

Unless otherwise specified, the following clinical features were defined according to the 2019 EULAR/ACR criteria for SLE (6). All manifestations needed to be present currently or no more than one month before the recording time. Cutaneous manifestations included acute cutaneous lupus, oral ulcer, non-scarring alopecia, and subacute cutaneous or discoid rash. Serositis included pleuritis and pericarditis. Nephritis needed a current proteinuria >0.5 g per day. Hematologic manifestations included hemolytic anemia, leukopenia (<4000/mm^3^), and thrombocytopenia (<100,000/mm^3^). The definitions of other clinical features were defined as follows: NP lupus was assessed according to the ACR nomenclature and case definitions for neuropsychiatric lupus (7). Pulmonary hypertension (PH) was diagnosed based on right heart catheterization or echocardiography (peak tricuspid regurgitation velocity >3.4 m/s). Gastrointestinal manifestations included protein-losing gastroenteropathy, mesenteric vasculitis, and colitis.

The disease duration was calculated from the diagnosis date for each case. Naive patients were defined as those cases within three months since SLE diagnosis and having no current or historical use of any systemic steroids, hydroxychloroquine, immunosuppressants, or targeted therapy.

### Immune tests

For immunological analysis, we required hospitalization records additionally on top of active SLE: the eligible records needed to have complete test results of anti-dsDNA antibody, serum complements, and lymphocyte subtype counting. All these tests were performed by the clinical laboratory of Renji hospital. All tests were clinical routine and, in most cases, performed within the first two days after admission. Anti-dsDNA antibody was detected by radioimmunoassay. The lymphocyte subset test was detected by a commercial kit (BD 340503) based on flow cytometry. It classified B cell, CD4+ T cell, CD8+ T cell, and NK cell from the total lymphocyte by the antigen combination of CD3 FITC, CD16 PE + CD56 PE, CD45 PerCP, and CD19 APC.

### Statistical methods

The Cochran-Armitage trend test and Cochran-Mantel-Haenszel test were employed to compare the presence of each clinical manifestation among groups with different disease duration. Serological test results/lymphocyte phenotype with disease duration was tested by the Spearman correlation. Multivariate linear regression was used to identify associators with lymphocyte phenotype. Wilcoxon test was used for comparison between parameters of repeated hospitalizations of one patient. A p-value of less than 0.05 was considered to be statistically significant. All statistical analyses were performed by SPSS (SPSS Inc., Chicago, IL, USA).

## Results

### Duration biased distribution of clinical phenotypes of active SLE

**Figure 1:**
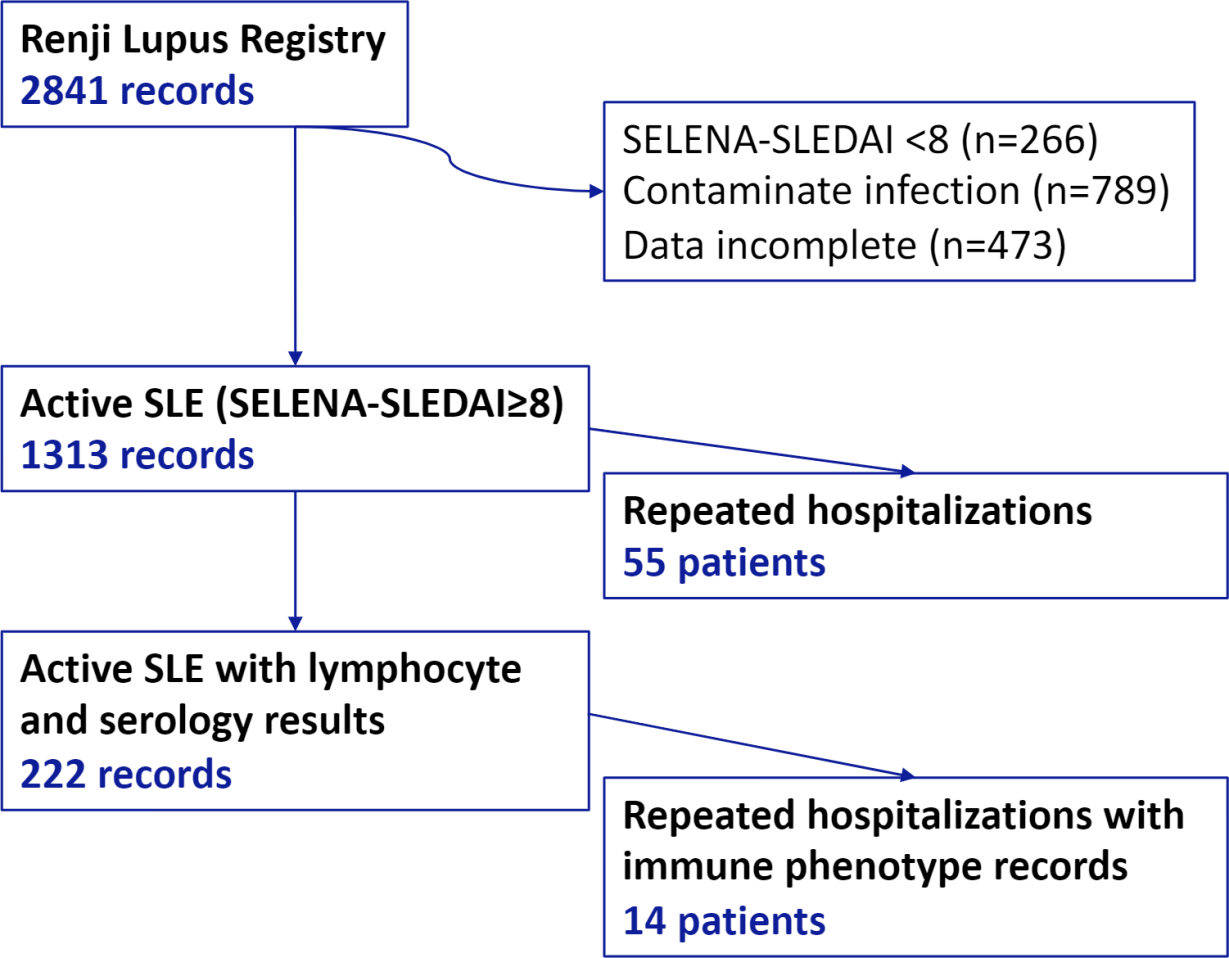
Study populations.

From 2013 to 2019, a total of 2032 patients with 2841 hospitalization records were enrolled in our registry. We enriched a total of 1313 eligible records on active SLE (SLEDAI-2k≥8) from the Renji Lupus registry, a registry study of hospitalized SLE patients with standard care (**Figure 1**). 92% of patients were female. The median age was 34 years old (IQR: 26-43). There were no statistical discrepancies among study populations. 65% of patients had recent conventional immunosuppressants (IS), and 4.4% of the patients received recent rituximab (**Table 1**). The study population was national-wide and covered 27/34 provinces of China. (**Supplementary Table 1**)

**Table 1:** Demographic and clinical features of the population.

	For clinic manifestations		For immunological phenotype		P value
	Total (1313 records, 1251 patients)	Repeated hospitalizations (55 patients)	Total (222 records, 207 patients)	Repeated hospitalizations (14 patients)	
Female	1118 (89.4%)	49 (89.1%)	190 (91.7%)	11 (78.6%)	0.4 ^a^
Age (years, median, IQR)	32 (24–42)	31 (22-40)	34 (26-43)	29 (20-45)	0.1939 ^b^
Age of disease onset (years, median, IQR)	26 (20-37)	23 (17-33)	27 (20-37)	24 (17-36)	0.0977 ^b^
Duration ^c^ (months, median, IQR)	40 (6-96)	61 (14-126)	48 (10-121)	30 (4-156)	0.0561 ^b^
SLEDAI (median, IQR)	9 (8-12)	10 (9-13)	10 (8-12)	10 (9-14)	0.2602 ^b^
Recent conventional IS use ^d^	767 (65.0%)	67 (60.9%)	148 (76.3%)	18 (64.3%)	0.13 ^a^
CYC	216 (18.3%)	18 (16.4%)	43 (22.2%)	9 (32.1%)	
MMF	234 (19.8%)	22 (20.0%)	41 (21.1%)	5 (17.9%)	
CNI	142 (12.0%)	11 (10.0%)	33 (17.0%)	4 (14.3%)	
AZA	63 (5.3%)	2 (1.8%)	14 (7.2%)	2 (7.1%)	
MTX	97 (8.2%)	7 (6.4%)	10 (5.2%)	1 (3.6%)	
Recent RTX use ^e^	48 (4.1%)	10 (9.1%)	4 (2.1%)	1 (3.6%)	0.04 ^a^
Antimalarial drug use	980 (83.1%)	93 (84.5%)	157 (80.9%)	17 (60.7%)	0.03 ^a^
Medication stop > 6 months ^f^	49/1120 (4.4%)	1/55 (1.8%)	11/194 (5.7%)	0/14 (0)	0.52 ^a^

^a^ Calculated by Fisher’s exact probability.

^b^ Calculated by one-way ANOVA.

^c^ Calculated from the SLE diagnosis date.

^d^ Any use of the following agents within three months until hospitalization.

^e^ Any use of rituximab within six months until hospitalization.

^f^ Medication stop was defined as any ever occurrence of no use of any systemic steroids, conventional IS, or targeted therapies. Antimalarial drug monotherapy was allowed.

IS, immunosuppressants; CYC, cyclophosphamide; MMF, mycophenolate mofetil; CNI, calcineurin inhibitor; AZA, azathioprine; MTX, methotrexate; RTX, rituximab.

**Figure 2:**
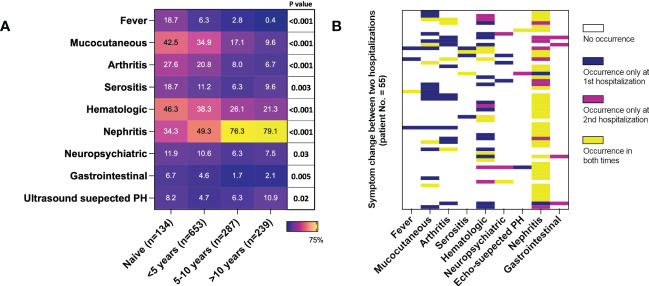
Duration biased clinical phenotype distribution in active SLE. 1313 cases were included. **(A)** Presence of SLE manifestations in groups of different disease durations. Each cell represents the occurrence of one symptom in the duration group in percentage. P values were calculated by the Cochran-Armitage trend test for each manifestation. **(B)** Changes in clinical manifestations of 55 patients with repeated hospitalizations due to active lupus (both hospitalizations with SLEDAI≥8). PH, pulmonary hypertension.

Divided by a 5-year interval of disease duration, the presence of fever, cutaneous symptoms, arthritis, hemocytopenia, neuropsychiatric (NP), and gastrointestinal manifestations descended in the long-duration groups, while nephritis and pulmonary hypertension ascended significantly (Cochran-Armitage trend test for each clinical dimension was significant, P values are illustrated in **Figure 2A**). Of the 239 active patients with SLE for more than 10 years, 79.1% had active nephritis, 21.3% had hemocytopenia, and other symptoms were 10% or less. In the subgroup analysis stratified with disease onset age, the trend of fever, mucocutaneous, arthritis, and hematological impairment were still significant in patients with younger onset ages (≤ 45 years old). The trend of nephritis was significant in all onset groups (**Supplementary Table 2**).

55 patients had repeated records with a median interval of 425 days (IQR: 233-834 days), and their longitudinal data supported the cross-sectional findings. Symptoms, including fever, arthritis, serositis, and cutaneous symptoms, were present on the first visit, but most of them did not occur in the second hospitalization. Besides, among patients without these symptoms during the first time, none of them had a new onset until the second hospitalization. On the contrary, most nephritis (44/46) was a flare or new onset in the second visit. Hematological impairment was mixed; 14 patients had it only the first time, and 12 patients only the second time. (**Figure 2B**)

### Duration biased distribution of immunological phenotypes of active SLE

**Figure 3:**
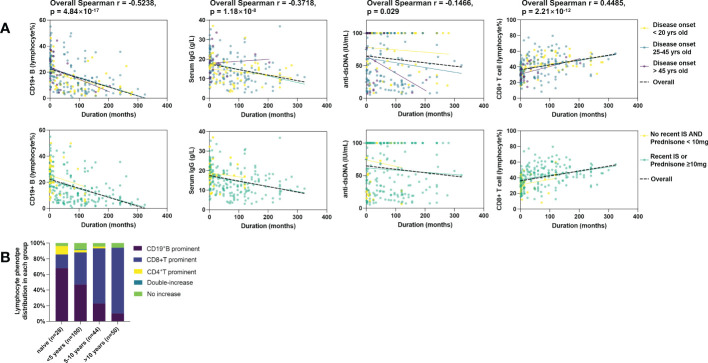
Correlation of immunological phenotypes of active SLE with disease duration. 222 cases were included. **(A)** Correlation of B cell%, serum IgG, serum anti-dsDNA, and CD8+ T cell% with disease duration. The black lines represent fit plots for the whole study population (n=222), and colored lines represent fit plots for subgroups by disease onset age (top) or recent treatment (bottom). **(B)** Duration biased distribution of lymphocyte balance in active SLE. The definition of lymphocyte types is shown in **Supplementary Table 5**.

222 records contained full results of serology and lymphocyte subset. The total number of lymphocytes was not correlated with duration. However, we observed a strong negative correlation between disease duration and peripheral B cell proportion and a simultaneously moderate positive correlation between disease duration and peripheral CD8+ T cell proportion. Moderate negative correlations with IgG and IgM were observed as well. Although a ceiling effect of anti-dsDNA was present in 39.2% of cases over the upper limit of radioimmunoassay detection, The negative correlation of dsDNA antibody with duration was still significant. The CD4+ T cells or NK cells were not correlated with disease duration. (**Table 2**) Sensitivity analysis showed consistent correlations in most subgroups stratified according to disease onset age or recent treatment (**Figure 3A**). Disease duration, age of disease onset, presence of recent steroids/IS, and recent use of CNI was associated with both B cell and CD8+ T cell proportions in the univariate analysis. (**Supplementary Table 3**) Only disease duration was significantly associated with both B and CD8+ T cell proportion in the multivariate analysis (**Table 3** and **Supplementary Table 4**).

**Table 2:** The correlation of disease duration with immune parameters (n=222).

	Spearman r	95% CI of r	P values
Total lymphocyte	-0.04591	-0.1803 to 0.09017	0.4961
B cell count	-0.4449	-0.5474 to -0.3292	**3.45×10^-12^**
B cell (lymphocyte%)	-0.5238	-0.6156 to -0.4179	**4.84×10^-17^**
anti-dsDNA	-0.1466	-0.2766 to -0.01128	**0.029**
IgG	-0.3718	-0.4832 to -0.2486	**1.18×10^-8^**
IgM	-0.4129	-0.5196 to -0.2935	**1.65×10^-10^**
IgA	-0.1022	-0.2347 to 0.03414	0.13
C3	0.1196	-0.01619 to 0.2510	0.0754
C4	0.2527	0.1214 to 0.3754	0.0001
CD8+T cell count	0.1481	0.01288 to 0.2781	**0.0273**
CD8+T cell (lymphocyte%)	0.4485	0.3332 to 0.5505	**2.21×10^-12^**
CD4+T cell count	-0.0406	-0.1752 to 0.09544	0.5473
CD4+T cell (lymphocyte%)	-0.00329	-0.1387 to 0.1323	0.9612
NK cell count	0.1202	-0.01556 to 0.2516	0.0739
NK cell (lymphocyte%)	0.1670	0.03223 to 0.2958	0.0127

**Table 3:** Multivariate analysis of associators for peripheral B cell and CD8+ T cell proportion in active SLE cases (n=222, Model 1).

	B cell (lymphocyte%)^a^					CD8+ T cell (lymphocyte%)^b^		
Factor	β	95% CI	Std. β	P value	β	95% CI	Std. β	P value
SLE onset age (years)	0.057	-0.073 to 0.186	0.056	0.39	-0.126	-0.274 to 0.021	-0.11	0.093
Duration (months)	-0.06	-0.081 to -0.038	-0.363	**8.9×10^-8^**	0.049	0.025 to 0.074	0.265	**7.6×10^-5^**
Female sex	2.374	-2.678 to 7.425	0.058	0.355	6.051	0.301 to 11.801	0.131	0.039
Recent Treatment^c^								
RTX	-2.16	-13.19 to 8.875	-0.024	0.7	2.158	-10.4 to 14.714	0.021	0.735
CNI	-3.42	-7.714 to 0.874	-0.099	0.118	4.452	-0.436 to 9.339	0.113	0.074
MTX	3.241	-3.921 to 10.402	0.056	0.373	-5.412	-13.56 to 2.74	-0.082	0.192
AZA	-3.75	-9.895 to 2.403	-0.076	0.231	3.93	-3.069 to 10.93	0.07	0.27
MMF	-2.44	-6.323 to 1.44	-0.078	0.216	4.218	-0.2 to 8.636	0.119	0.061
CTX	-0.25	-4.044 to 3.544	-0.008	0.897	3.848	-0.47 to 8.167	0.111	0.08

a R^2^ = 0.211.

b R^2^ = 0.208.

c Any use of the conventional immunosuppressants within three months or rituximab within six months until hospitalization.

CYC, cyclophosphamide; MMF, mycophenolate mofetil; CNI, calcineurin inhibitor; AZA, azathioprine; MTX, methotrexate; RTX, rituximab.

The balance of lymphocyte subsets was defined by the reference intervals of each subset for the Chinese population (Detailed definitions are in **Supplementary Table 5**) (8). As shown in **Figure 3B**, the B cell prominent and CD8+T cell prominent were the two major phenotypes, while other types together less than 15.3%. No NK-prominent type was found. In consistency with the correlation findings, the frequency of the B prominent type declined in duration groups, and the CD8 prominent type arose opposite to the B type with disease duration (Fisher’s exact probability=7.76×10^-9^). In the patients with a duration of over ten years, only 10% of patients still had B cell elevated, and up to 84% of patients were CD8 type.

**Figure 4:**
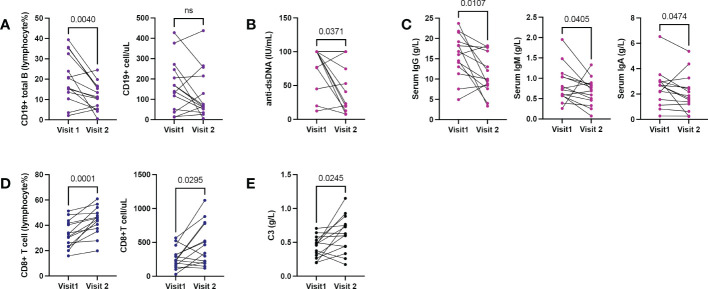
Shifting immune phenotypes in longitudinal records of active SLE. 14 patients with repeated hospitalizations due to active lupus (each hospitalization with SLEDAI≥8). **(A)** Changes in B cell counts and proportions. **(B, C)** Changes in serum anti-dsDNA and serum immunoglobulin levels. **(D)** Changes in CD8+T cell counts and proportions. **(E)** Changes in serum C3. Wilcoxon-test for all comparisons.

Fourteen patients had repeated hospitalizations with lymphocyte subset records. The median interval was 306 days (range: 84-672 days). Compared to the first hospitalization, B cell proportion (**Figure 4A**), anti-dsDNA (**Figure 4B**), and serum IgG (**Figure 4C**) decreased significantly while the CD8+ T population (**Figure 4D**) and serum C3 (**Figure 4E**) increased in the second visit.

## Discussion

To our knowledge, this study is the first systematic description of clinical and immune features of SLE patients in long-term duration. Catching clinical and immunological features of active disease for established patients (i.e., flares or refractory activity) in a large number is not easy for patients usually with low activity in long-term follow-up, even in a large cohort (9). Our study somehow overcame this difficulty and provided a large number of patients with active disease and long-duration *via* enriched patients with SLEDA-2k≥8 from a big registry. Moreover, we extracted longitudinal datasets from the whole study population as confirmations, which we believed to the largest extent compensated the shortness of a cross-sectional study.

Previous reports on the shifting clinical phenotypes were fragmented (10). Among these, lupus nephritis was the most reported organ involvement increased with disease duration (9, 11–13). Recently, a decrease in NP events with lupus duration was observed (14). In consistence with previous observations that hematological impairment was a common flare (11–13), we for the first time show the mixed manner of hematological impairment (**Figure 2B**), which could be led by different pathogenetic ways. Arthritis and cutaneous flare were common in short follow-up (12, 13), but this study offered an opposite view of long-established SLE activation. PH of SLE varies from association with disease activity in SLE, response to immunosuppressive therapy, and presence in early or established patients (15, 16). The ascending trend of PH is reported for the first time in this study and is worthy of further investigation.

This work highlights the impact of disease duration on lupus immune phenotype. Along with disease evolution, SLE activates with a seemly dampened humoral immune response, with a parallel decrease in peripheral B cell, anti-dsDNA, and serum IgG and IgM. Lower anti-dsDNA antibody level in late-onset lupus nephritis patients (17), and a seemly decrease in ANA positive rate in a 5-year follow-up of SLE were reported as secondary results previously (9).

This duration-associated dampened humoral immune response may further lead to a discrepant response to B-cell-targeted therapies. Recently, a small study found that SLE patients with longer durations had difficulties achieving complete depletion of peripheral B cells (18), and a small observation shows that a shorter duration of LN responds better to rituximab (19). Our findings merits more detailed profiling of B cell in duration courses, especially the balance of physiological/pathological B cells, which may give a better interpretation of this phenomenon.

Consistent with a recent finding that circulating cytotoxic CD8+ T cell expended in SLE patients (20), a duration-associated relative increase of CD8+ T cells is observed for the first time in our study. The previous functional studies on CD8+T cells in SLE focused on their impaired cytotoxicity, maintenance of autoimmunity, and the damaging role of tissue CD8+T cells (21). Whether the CD8+ T increase is secondary or pathological needs further investigation.

The highlighted disease duration in this study implies two sub-factors, treatment and aging, both of which are well-known impactors on the immune system. To analyze the impact of treatment, we separated groups of naive patients from those with disease duration < 5 years as a reference. Besides, the overall immunosuppressive treatment, as well as some single agents, were associated with peripheral B cell% and CD8 T cell% in univariate analysis. Regarding aging, we used the age of SLE onset to adjust its impact on disease duration, and onset age was also associated with peripheral B cell% and CD8 T cell% in univariate analysis. However, in multivariate analysis, neither overall treatment nor onset age was significant. Therefore, we believe it is reasonable to consider duration as a single factor when assessing the pathogenesis evolution in SLE.

The limitations of this study are the following: 1) This study is limited to hospitalized patients, which may underestimate some symptoms routinely treated in the clinics, such as chronic cutaneous lupus and arthritis (22, 23). 2) We did not have the information in this registry on exactly when each patient fulfilled the SLE classification criteria. Therefore, we calculated the disease duration alternatively from the date of SLE diagnosis, which could underestimate the disease duration. 3) There was a large number of missing cases in the immune type analysis. This was because we required patients with lymphocyte subtype test results, as well as other serum markers if they could be enrolled, for we wanted the serological test matched with cell types. However, the lymphocyte subtype test (BTNK) was not a clinical routine in our hospital until about 2017, and we had to exclude most cases before 2017.

## Conclusion

This study reveals a comprehensive picture of clinical and immunological features of active SLE patients, which is significantly duration biased. Disease duration should be considered as an important confounder in future investigations on SLE.

## Data availability statement

The data analyzed in this study is subject to the following licenses/restrictions: All data are from the Renji Lupus Registry, an internal medical system of Renji Hospital, Shanghai Jiao Tong University School of Medicine. Requests to access these datasets should be directed to QY, yanqingran@renji.com. We can provide data excel sheets, but the data is not uploaded, all without links or websites.

## Ethics statement

The study was conducted in accordance with the Declaration of Helsinki and was approved by the Ethics Committee of Renji Hospital, Shanghai, China (ID: 2013-126). The patients/participants provided their written informed consent to participate in this study.

## Author contributions

QY, BL, MY contributed equally to this work and share the first authorship. QT, BL, and MY carried out the study with support from QL and BL. QY and LL initiated and designed the project. TL built the registrydataset. JW provided statistical support. QY wrote the manuscript. All authorsdiscussed the results and contributed to the final manuscript. All authors read and approved the final manuscript.
