# Supplementary material for: Duration biased distribution of clinical and immunological phenotypes in active SLE
Source: Front Immunol. 2022 Dec 14;13:1044184. doi: 10.3389/fimmu.2022.1044184 (PMC9794596; doi:10.3389/fimmu.2022.1044184)
Supplement: Supplementary file 1 [file DataSheet_1.docx]

**Supplementary Table 1. Geographic distribution of the study population**

| Province* | Patient No. | Proportion (%) |
| --- | --- | --- |
| Anhui | 128 | 10.20 |
| Beijing | 6 | 0.48 |
| Fujian | 59 | 4.75 |
| Gansu | 12 | 0.93 |
| Guangdong | 8 | 0.64 |
| Guizhou | 25 | 2.02 |
| Hebei | 12 | 0.93 |
| Henan | 66 | 5.29 |
| Heilongjiang | 53 | 4.20 |
| Hubei | 66 | 5.29 |
| Hunan | 18 | 1.48 |
| Jilin | 3 | 0.24 |
| Jiangsu | 128 | 10.20 |
| Jiangxi | 87 | 6.93 |
| Liaoning | 18 | 1.48 |
| Nei Mongol | 4 | 0.24 |
| Ningxia | 12 | 0.93 |
| Qinghai | 2 | 0.16 |
| Shandong | 53 | 4.20 |
| Shaanxi | 12 | 0.93 |
| Shanxi | 4 | 0.32 |
| Shanghai | 203 | 16.20 |
| Sichuan | 39 | 3.11 |
| Xinjiang | 12 | 0.93 |
| Yunnan | 25 | 2.02 |
| Chongqing | 12 | 0.93 |
| Zhejiang | 134 | 10.74 |
| Unknown | 52 | 4.24 |
| Total | 1251 |  |

* Our population covered 27/34 Provinces of China.

**Supplementary Table 2. Stratified analysis of clinical manifestation and disease duration in active SLE cases (n=1313).**

|  |  | Duration since diagnosis | | | |  |
| --- | --- | --- | --- | --- | --- | --- |
| Symptoms | Age of onset/years | Naive | <5 years | 5-10 years | >10 years | P value^b^ |
| Fever | <20 | 5(20.8%)^a^ | 11(7.6%) | 3(4.0%) | 0 | 0.001 |
|  | 20-45 | 16(18.0%) | 21(5.1%) | 5(2.6%) | 1(0.7%) | <0.001 |
|  | >45 | 4(19.0%) | 9(9.1%) | 0 | 0 | 0.068 |
|  | total | 25(18.7%) | 41(6.3%) | 8(2.8%) | 1(0.4%) | <0.001 |
| Mucocutaneous | <20 | 11(45.8%) | 62(43.1%) | 18(24.0%) | 9(10.7%) | <0.001 |
|  | 20-45 | 39(43.8%) | 137(33.4%) | 30(15.6%) | 13(8.8%) | <0.001 |
|  | >45 | 7(33.3%) | 29(29.3%) | 1(5.0%) | 1(14.3%) | 0.1 |
|  | total | 57(42.5%) | 228(34.9%) | 49(17.1%) | 23(9.6%) | <0.001 |
| Arthritis | <20 | 5(20.8%) | 28(19.4%) | 3(4.0%) | 3(3.6%) | <0.001 |
|  | 20-45 | 28(31.5%) | 85(20.7%) | 18(9.4%) | 12(8.1%) | <0.001 |
|  | >45 | 4(19.0%) | 23(23.2%) | 2(10.0%) | 1(14.3%) | 0.524 |
|  | total | 37(27.6%) | 136(20.8%) | 23(8.0%) | 16(6.7%) | <0.001 |
| Serositis | <20 | 3(12.5%) | 17(11.8%) | 3(4.0%) | 10(11.9%) | 0.262 |
|  | 20-45 | 18(20.2%) | 48(11.7%) | 13(6.8%) | 12(8.1%) | 0.005 |
|  | >45 | 4(19.0%) | 8(8.1%) | 2(10.0%) | 1(14.3%) | 0.551 |
|  | total | 25(18.7%) | 73(11.2%) | 18(6.3%) | 23(9.6%) | 0.002 |
| Hematologic | <20 | 10(41.7%) | 55(38.2%) | 18(24.0%) | 19(22.6%) | 0.027 |
|  | 20-45 | 41(46.1%) | 155(37.8%) | 51(26.6%) | 31(20.9%) | <0.001 |
|  | >45 | 11(52.4%) | 40(40.4%) | 6(30.0%) | 1(14.3%) | 0.223 |
|  | total | 62(46.3%) | 250(38.3%) | 75(26.1%) | 51(21.3%) | <0.001 |
| Nephritis | <20 | 5(20.8%) | 71(49.3%) | 61(81.3%) | 64(76.2%) | <0.001 |
|  | 20-45 | 35(39.3%) | 212(51.7%) | 143(74.5%) | 120(81.1%) | <0.001 |
|  | >45 | 6(28.6%) | 39(39.4%) | 15(75.0%) | 5(71.4%) | 0.005 |
|  | total | 46(34.3%) | 322(49.3%) | 219(76.3%) | 189(79.1%) | <0.001 |
| Neuropsychiatric | <20 | 5(20.8%) | 21(14.6%) | 4(5.3%) | 7(8.3%) | 0.068 |
|  | 20-45 | 8(9.0%) | 40(9.8%) | 14(7.3%) | 10(6.8%) | 0.62 |
|  | >45 | 3(14.3%) | 8(8.1%) | 0 | 1(14.3%) | 0.206 |
|  | total | 16(11.9%) | 69(10.6%) | 18(6.3%) | 18(7.5%) | 0.094 |
| Gastrointestinal | <20 | 2(8.3%) | 7(4.9%) | 1(1.3%) | 3(3.6%) | 0.382 |
|  | 20-45 | 6(6.7%) | 17(4.1%) | 4(2.1%) | 2(1.4%) | 0.089 |
|  | >45 | 1(4.8%) | 6(6.1%) | 0 | 0 | 0.396 |
|  | total | 9(6.7%) | 30(4.6%) | 5(1.7%) | 5(2.1%) | 0.022 |
| Ultrasound suspected PH | <20 | 1(4.2%) | 7(4.9%) | 3(4.0%) | 12(14.3%) | 0.027 |
|  | 20-45 | 9(10.1%) | 19(4.6%) | 14(7.3%) | 13(8.8%) | 0.128 |
|  | >45 | 1(4.8%) | 5(5.1%) | 1(5.0%) | 1(14.3%) | 0.847 |
|  | total | 11(8.2%) | 31(4.7%) | 18(6.3%) | 26(10.9%) | 0.01 |

^a^ The cell is presented as the number of cases (percentage of fever cases/naive cases).

^b^ Calculated by Cochran-Mantel-Haenszel test.

PH, pulmonary hypertension.

**Supplementary Table 3. Univariate analysis of associators for B cell% and CD8+ T cell% in active SLE cases (n=222)**

| **Univariate analysis for CD19+ B cell (lymphocyte%)** | | | | |  |  |
| --- | --- | --- | --- | --- | --- | --- |
| Factor | β | 95% CI | | | Std. β | P value |
| Age (years) | -0.064 | -0.194 to 0.065 | | | -0.066 | 0.329 |
| Age of disease onset (years) | 0.156 | 0.023 to 0.288 | | | 0.154 | 0.022 |
| Sex | 0.982 | -4.439 to 6.404 | | | 0.024 | 0.721 |
| Duration (months) | -0.067 | -0.087 to -0.048 | | | -0.421 | 5.612×10^-11^ |
| Recent treatment^a^ |  |  |  |  | |  |
| RTX | 1.9 | -10.142 to 13.94 | | | 0.021 | 0.756 |
| MMF | -2.306 | -6.422 to 1.809 | | | -0.074 | 0.271 |
| CTX | 0.759 | -3.294 to 4.811 | | | 0.025 | 0.712 |
| CNI | -5.673 | -10.112 to -1.234 | | | -0.167 | 0.012 |
| AZA | -5.417 | -11.968 to 1.135 | | | -0.109 | 0.105 |
| MTX | 2.854 | -4.861 to 10.57 | | | 0.049 | 0.467 |
| No recent IS AND Prednisone < 10mg^b^ | 6.867 | 1.969 to 11.77 | | | 0.183 | 0.006 |
| **Univariate analysis for CD8+ T cell (lymphocyte%)** | | | | |  |  |
| Factor | β | 95% CI | | | Std. β | P value |
| Age (years) | 0.03 | -0.117 to 0.178 | | | 0.027 | 0.685 |
| Age of disease onset (years) | -0.182 | -0.332 to -0.031 | | | -0.158 | 0.018 |
| Sex | 6.948 | 0.86 to 13.04 | | | 0.15 | 0.025 |
| Duration (months) | 0.064 | 0.042 to 0.087 | | | 0.354 | 5.686 × 10^-8^ |
| Recent treatment^a^ |  |  |  |  | |  |
| RTX | -0.964 | -14.64 to 12.71 | | | -0.009 | 0.89 |
| MMF | 3.4 | -1.264 to 8.064 | | | 0.096 | 0.152 |
| CTX | 2.891 | -1.7 to 7.481 | | | 0.083 | 0.216 |
| CNI | 6.759 | 1.726 to 11.79 | | | 0.175 | 0.009 |
| AZA | 5.106 | -2.346 to 12.56 | | | 0.09 | 0.178 |
| MTX | -6.074 | -14.807 to 2.66 | | | -0.092 | 0.172 |
| No recent IS AND prednisone < 10mg^b^ | -7.19 | -12.766 to -1.615 | | | -0.169 | 0.012 |

^a^ Any use of the conventional immunosuppressants within three months or rituximab within six months until hospitalization.

^b^ No use of conventional immunosuppressants and daily prednisone <10mg within three months, and rituximab within six months until hospitalization.

IS, immunosuppressants; CYC, cyclophosphamide; MMF, mycophenolate mofetil; CNI, calcineurin inhibitor; AZA, azathioprine; MTX, methotrexate; RTX, rituximab.

**Supplementary Table 4. Multivariate analysis of associators for peripheral B cell and CD8+ T cell proportion in active SLE cases (n=222, Model 2).**

|  | B cell (lymphocyte%)^a^ | | | | | CD8+ T cell (lymphocyte%)^b^ | | | |
| --- | --- | --- | --- | --- | --- | --- | --- | --- | --- |
| Factor | β | 95% CI | Std. β | P value | β | | 95% CI | Std. β | P value |
| SLE onset age (years) | 0.058 | -0.07 to 0.185 | 0.057 | 0.365 | -0.13 | | -0.28 to 0.016 | -0.113 | 0.081 |
| Female sex | 1.919 | -3.1 to 6.934 | 0.047 | 0.452 | 6.695 | | 0.891 to 12.498 | 0.144 | 0.024 |
| Duration (months) | -0.06 | -0.08 to -0.043 | -0.393 | <0.0001 | 0.054 | | 0.031 to 0.078 | 0.298 | <0.0001 |
| No recent IS AND Prednisone < 10mg^c^ | 3.673 | -0.93 to 8.28 | 0.098 | 0.117 | -4.262 | | -9.59 to 1.069 | -0.1 | 0.117 |

^a^ R^2^ = 0.193.

^b^ R^2^ = 0.162.

^c^ No use of conventional immunosuppressants and daily prednisone <10mg within three months, and rituximab within six months until hospitalization.

IS, immunosuppressants.

**Supplementary Table 5. Definition of lymphocyte subset phenotypes.**

|  | Lymphocyte subset phenotype | | | | | Reference interval  (P2.5–P97.5) | |
| --- | --- | --- | --- | --- | --- | --- | --- |
| Subset | B prominent | CD4+ T prominent | CD8+ T prominent | No increase | Double increased | Lower (%) | Upper (%) |
| CD3+CD4+ | —/↓ | ↑ | —/↓ | —/↓ | Any two subsets over the upper limits simultaneously | 22.2 | 50.25 |
| CD3+CD8+ | —/↓ | —/↓ | ↑ | —/↓ |  | 14.19 | 43.41 |
| CD3-CD19+ | ↑ | —/↓ | —/↓ | —/↓ |  | 5.05 | 20.45 |

↑, the subset percent of lymphocytes is over the upper limit of reference interval(1); —, the subset percent of lymphocytes is within the reference interval; ↓, the subset percent of lymphocytes is below the lower limit of reference interval.

Ref.

1. Xu K, Miao L, Chen W, Wu H, Gong Y, Tu X, et al. Establishment of the reference intervals of lymphocyte subsets for healthy Chinese Han adults and its influencing factors. Annals of translational medicine. 2021;9(19):1495-.
